# Gigahertz Frame Rate Imaging of Charge-Injection Dynamics in a Molecular Light Source

**DOI:** 10.1021/acs.nanolett.1c00328

**Published:** 2021-05-26

**Authors:** Anna Rosławska, Pablo Merino, Christopher C. Leon, Abhishek Grewal, Markus Etzkorn, Klaus Kuhnke, Klaus Kern

**Affiliations:** † Max-Planck-Institut für Festkörperforschung, D-70569 Stuttgart, Germany; ‡ Université de Strasbourg, CNRS, IPCMS, UMR 7504, F-67000 Strasbourg, France; § Instituto de Ciencia de Materiales de Madrid, CSIC, E-28049 Madrid, Spain; ∥ Instituto de Física Fundamental, CSIC, E-28006 Madrid, Spain; ⊥ Institut für Angewandte Physik, TU Braunschweig, D-38106 Braunschweig, Germany; # Institut de Physique, École Polytechnique Fédérale de Lausanne, CH-1015 Lausanne, Switzerland

**Keywords:** Scanning tunneling microscopy-induced luminescence, nanosecond imaging, charge injection, charge dynamics

## Abstract

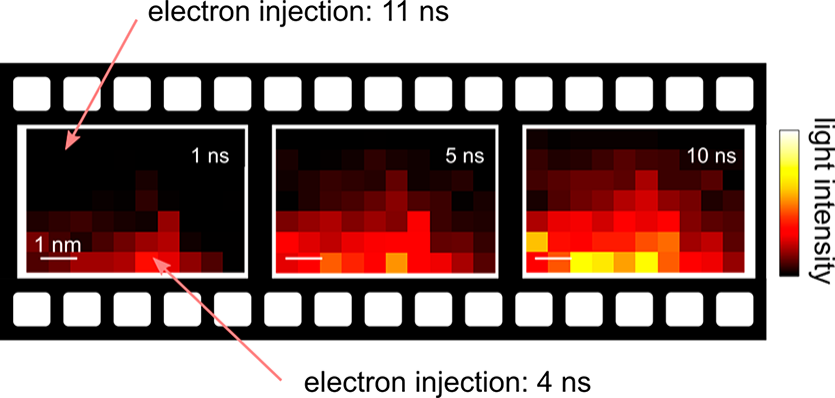

Light sources on the scale of single molecules can be addressed and characterized at their proper sub-nanometer scale by scanning tunneling microscopy-induced luminescence (STML). Such a source can be driven by defined short charge pulses while the luminescence is detected with sub-nanosecond resolution. We introduce an approach to concurrently image the molecular emitter, which is based on an individual defect, with its local environment along with its luminescence dynamics at a resolution of a billion frames per second. The observed dynamics can be assigned to the single electron capture occurring in the low-nanosecond regime. While the emitter’s location on the surface remains fixed, the scanning of the tip modifies the energy landscape for charge injection into the defect. The principle of measurement is extendable to fundamental processes beyond charge transfer, like exciton diffusion.

Energy conversion in both artificial and natural systems proceeds via a sequence of fundamental processes occurring at the quantum level of single photons and single electrons. The individual charges during redox reactions, biosynthesis, and light emission from optoelectronic devices undergo a series of processes like tunneling, hopping, or recombination leading to measurable chemical, electronic, or optical signals.^1,2^ In particular, the tunneling of the electron through short molecular bridges plays a crucial role in electron transport within molecular wires^3^ and between nucleic acids.^1^ While these charge transfer processes can be of a few picoseconds or less, they are typically an order of magnitude slower^2,4^ when the transfer distances are increased to a few nanometers and are thus accessible at the sub-nanosecond temporal scale and controllable at the sub-nanometer spatial scales.

Probing charged species with such spatial control can be achieved using scanning tunneling microscopy (STM). This approach is sensitive to the electronic density of states which enables electronic spectroscopy and imaging of charged single atoms,^5^ molecules,^6^ and defects,^7−10^ including elucidation of intramolecular details if combined with atomic force microscopy.^11^ These studies, however, investigated static systems, in which a charge was either permanently residing in the system or replenished faster than the time resolution of the measurement. Requiring the measurement of small currents in the pA range, the temporal resolution of STM is typically limited to millisecond resolution. This limitation can be overcome by using advanced methodologies like all-electronic pump–probe spectroscopy^12^ or coupling with ultrafast laser pulses.^13−15^ In such experiments, the signal is detected by employing the STM tunnel current to read out the averaged response of the system that varies with the delay between the applied pulses. Employing STM-induced luminescence (STML)^16^ in contrast provides a specific selectivity to processes that result in photon emission, in particular to electroluminescence of molecular emitters,^16−27^ including charged species,^17,18^ and allows steady-state submolecular mapping of electroluminescence.^20−23^ Thanks to time-resolved single-photon detectors, the STML signal can be probed with sub-nanosecond temporal resolution,^25,26,28^ albeit limited to local point measurements. In our work, we map the electroluminescence in the time-domain and record optical nanometer–nanosecond snapshots of light emitted by single defects in thin organic films that light up within a few nanoseconds after pulsed electronic excitation. Because photon emission is intimately linked to electron injection, the electroluminescence delay can be used as a real-time and real-space monitor of the occurrence of an individual nanosecond electron transfer process. The short lifetime of the intermediate singlet excitonic state (<1 ns), which converts the charge injection into light emission, preserves the time resolution of the measuring principle. The injection rate depends on the position of the STM tip, which remote-controls the electric field at the defect.

The experiment is schematically presented in Figure 1a. We study time-resolved STML (TR-STML) from individual defects in thin C_60_ films^22,26,27,29,30^ grown on a Au(111) substrate using a cryogenic (4 K) STM with optical access. The light emission from the defect is periodically induced by high-fidelity 100 ns long square pulses^12,31,32^ with sharp edges (∼1 ns rise time) and an amplitude *U*_pulse_ added to the static bias voltage. The response of the system is probed by recording TR-STML intensity transients, *P*(*t*), with sub-ns time-resolution (see the Methods section for more details), which reveal an exponential rise and decay with respect to the applied square pulse both encoding the time (τ_e_) a single electron takes to be captured in the defect from the substrate after a hole has been injected from the tip.^27^ This approach relies only on the optical signal and does not require a peculiar electronic configuration to access charge-injection rates.^31,33^

**Figure 1:**
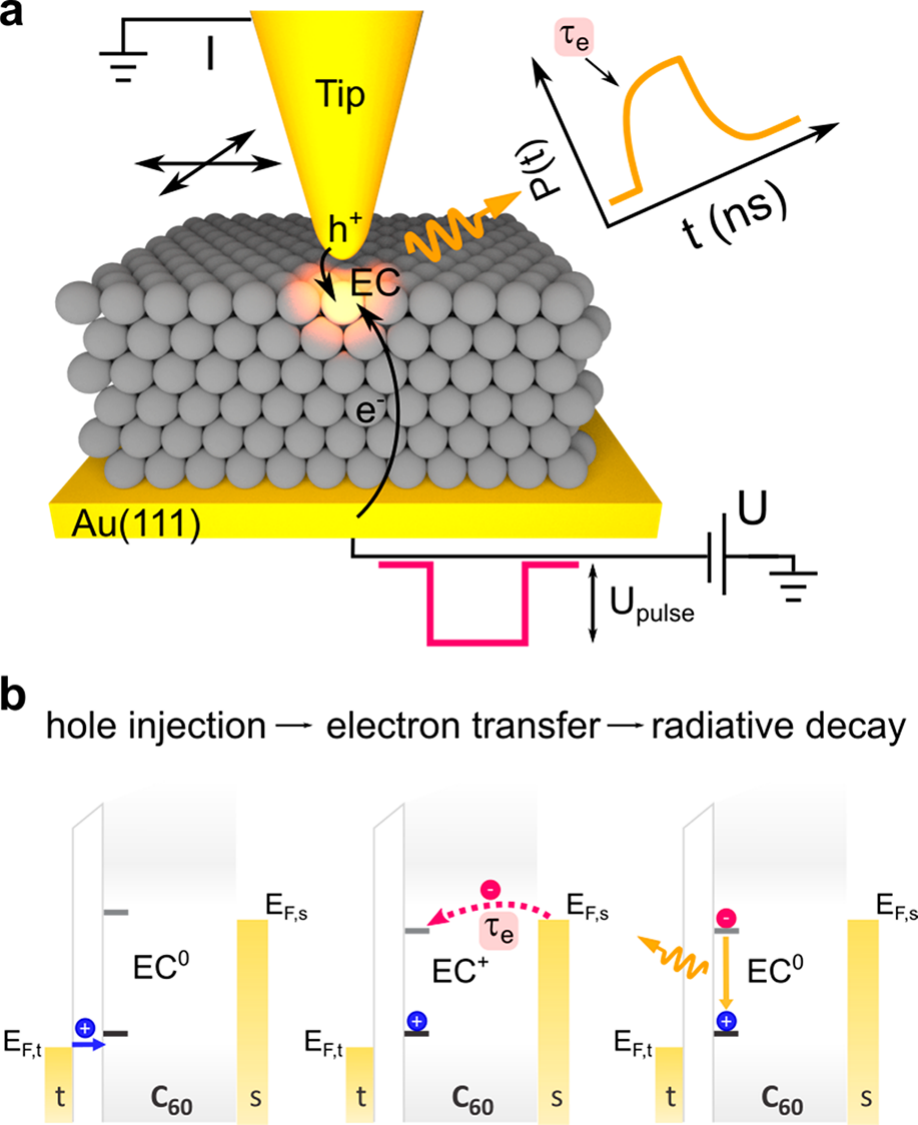
Probing the single electron injection dynamics. (a) Experimental scheme. Negative nanosecond voltage pulses (100 ns long, 1 ns rise/fall time) are applied to an STM junction. The pulses enable photon emission from an individual emission center (EC) in a C_60_ thin film, which is recorded as a function of the delay with respect to the pulse arrival. The measured exponential onset is a probe of the single electron injection time (τ_e_). By varying the tip position on the surface, we map τ_e_ with nm spatial precision. (b) Energy diagrams illustrating the mechanism of the electroluminescence, which is a result of a sequence of events labeled on top of the panel. t = tip, s = Au substrate.

In a first step, the geometry of the defect and its neighborhood are studied by STM topography. C_60_ molecules are resolved internally revealing their individual orientation in the top layer of the thin (<10 nm) film (Figure 2a). The simultaneously recorded electroluminescence yield at each pixel (photon map, Figure 2b) shows the spatially confined emission center (EC) localized around the molecule numbered as 1. It is known from earlier studies that ECs in C_60_ are related to structural defects that trap a hole and an electron and enable emission, otherwise symmetry forbidden, from the lowest singlet electron–hole state (exciton) of C_60_^22,26,27,29,30^ (Figure 2c). The optical emission spectra at an increasing distance from the EC, measured on molecules with identical orientation, still show the characteristic emission lines of C_60_ (Figure 2c).^22^ The origin of this specific EC is further discussed in the Supporting Information.

**Figure 2:**
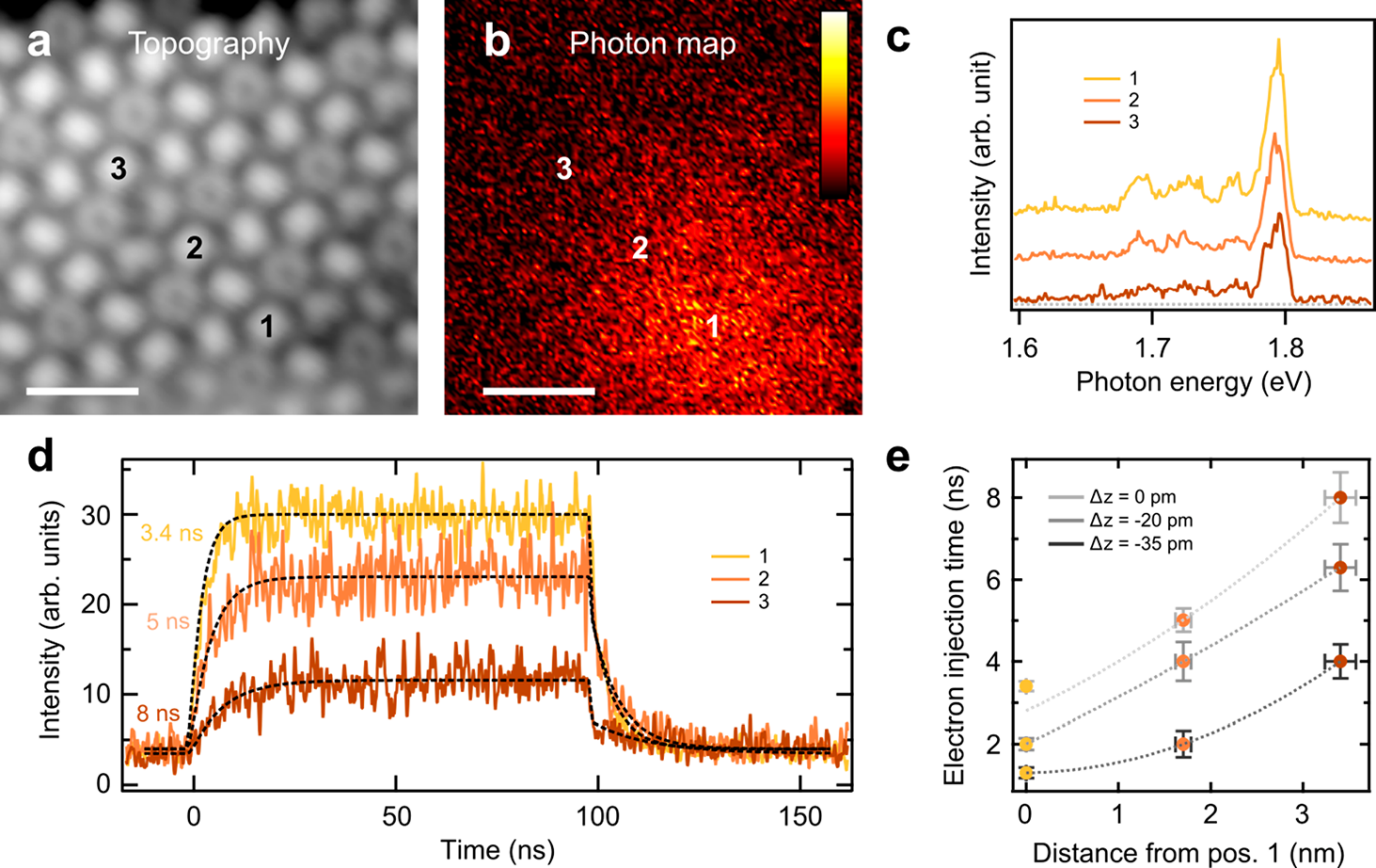
Electron transfer time to the defect as a function of the lateral tip position. (a) Constant current STM topography *U* = −3 V, *I* = 30 pA, scale bar 2 nm. (b) Electroluminescence yield map (photon map) recorded simultaneously with panel a. Color scale bar: 0–60 counts/(s pA). (c) Optical spectra recorded at positions marked in panels a and b. *U* = −3 V, *I* = 30 pA. The traces are offset for clarity; the dashed line indicates the baseline. (d) TR-STML transients measured at positions marked in panels a and b, average current during the pulse, *I*_pulse_ = 12 pA, *U*_on_ = −2.83 V, *U*_off_ = −2.53 V. The dashed lines represent fits to the kinetic model. The extracted τ_e_ values are indicated next to the traces. (e) Electron injection time measured at different horizontal positions (1–3) and different vertical offsets (Δ*z*). *I*_pulse_ = 12 pA (Δ*z* = 0 pm), *I*_pulse_ = 20 pA (Δ*z* = −20 pm), *I*_pulse_ = 37 pA (Δ*z* = −35 pm). The dashed lines are guides to the eye. The vertical error bars are the fitting errors. The horizontal error bar is 5% error of the distance measurement.

When the bias voltage of the STM is driven more negative by the transient voltage pulse than the applied static negative voltage, it enables hole injection into a neutral defect state (EC^0^) (left panel in Figure 1b) such that it becomes transiently charged (EC^+^). This defect is then neutralized by a single electron transfer from the Au(111) substrate, which occurs within time τ_e_ (middle panel in Figure 1b). Note that the electron transfer from the substrate is substantially enhanced by the strong electrostatic potential of the trapped hole which shifts the electron defect level below the Fermi energy of the substrate (*E*_F,s_).^27^ This process results in the creation of an electron–hole pair (exciton) at the defect that may decay radiatively by emitting a photon (right panel in Figure 1b). In this study, we apply voltage pulses of amplitude *U*_pulse_ that move the Fermi level of the tip (*E*_F,t_) from inside the band gap between the states derived from highest occupied molecular orbital (HOMO) and the lowest unoccupied molecular orbital (LUMO) of the C_60_ film to the highest lying HOMO states within 1 ns, switching on charge injection and subsequent photon emission.

In the next step, we study in detail the dynamics of this electron injection process as a function of lateral position. We choose 3 molecules (numbered 1–3) with identical orientation (hexagon–hexagon bond facing upward) where the same local density of states (LDOS) results in same tip height for a fixed tunneling current set point. This is particularly critical for comparing the respective dynamics at the molecules since the electron transfer rate depends strongly on the tip–sample distance.^27^ For consistency, we show that the emission spectra are identical at all three positions (Figure 2c) exhibiting no plasmonic contribution^30,34^ and varying only in intensity. We record the electroluminescence transients at the marked positions and plot them in Figure 2d. As observed directly from the TR-STML signal, the dynamics encoded in the rising and falling edges of the light pulse slow down when hole injection from the tip occurs farther from the center of the EC. Here, we would like to stress that the exciton recombination and photon emission are believed to always occur at the center of the EC, close to molecule 1. Only at this position are the selection rules relaxed, and the emission is permitted.^22,29^ The exciton lifetime remains shorter than 1 ns, and the hole injection is comparatively slow (μs regime) due to both the tunneling current and trapping efficiency being low,^26,27^ such that the majority of the current passing through the system does not contribute to the luminescence. Because these processes occur at time scales that are different from the one observed in the experiment, the dynamics observed in the luminescent transients can be related to the electron injection from the substrate. Its rate can be obtained by fitting the transient to a kinetic model describing the sequence shown in Figure 1b.^27,28^ In Figure 2e we plot the extracted τ_e_ as a function of the distance from the central molecule (1) and find that it increases from 3 to 8 ns when moving away laterally by 4 nm. Additionally, in line with our previous observations, we find that τ_e_ decreases when the tip–sample distance is reduced (three curves in Figure 2e).^27^ This can be ascribed to a reduction of the energy barrier at the C_60_/Au(111) interface due to the increase of the electrical field.

Next, we extend our analysis by mapping the charge-injection times as a function of the tip position near an EC by measuring TR-STML transients on a 10 × 7 point grid (see the Methods section). In Figure 3a, we plot a spatially interpolated map of extracted τ_e_, and in Figure 3b, we present the photon map to compare the spatial extension of the EC. As observed in Figure 2d, the electron injection time to EC^+^ increases when the tip is located at the periphery of the EC.

**Figure 3:**
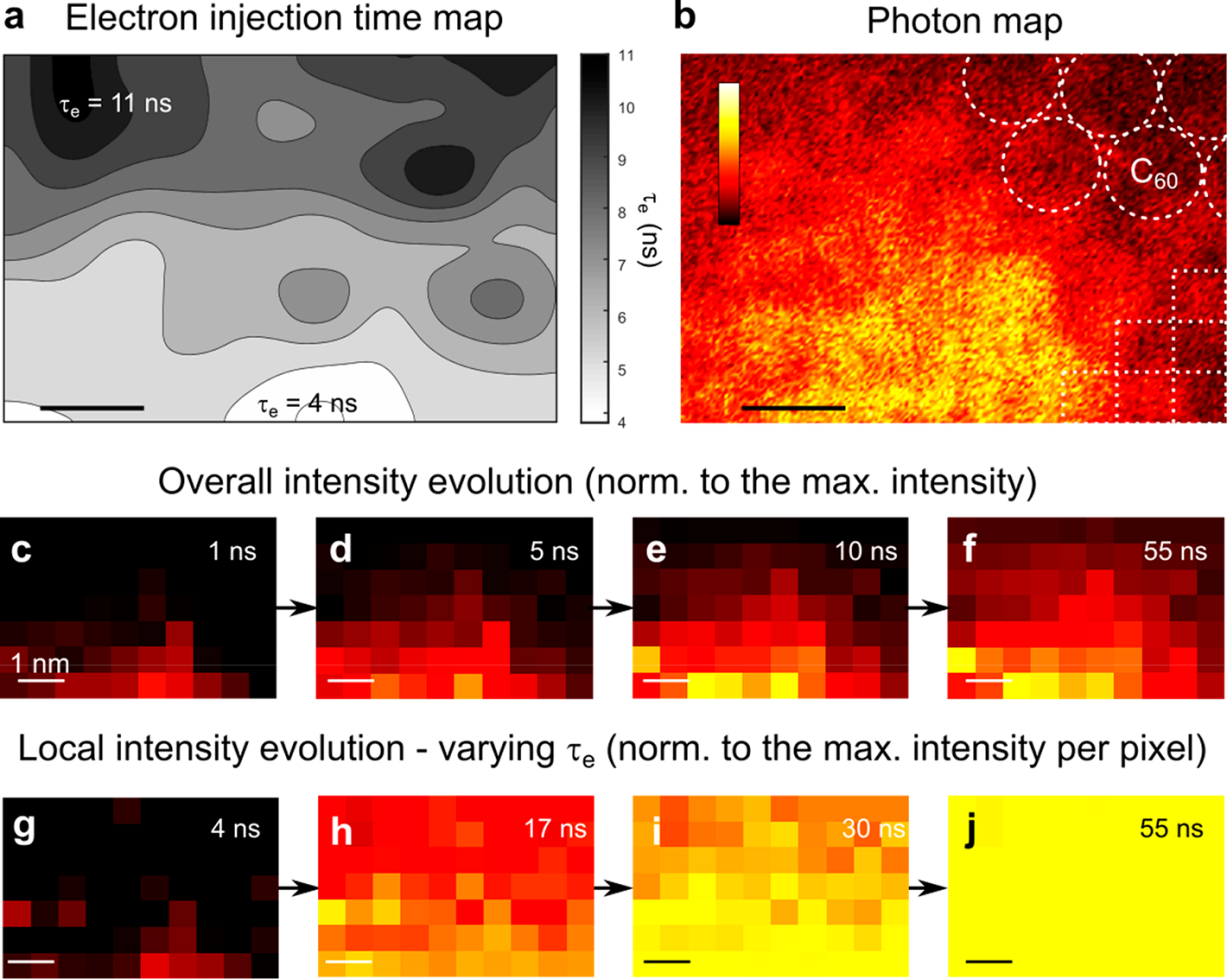
Electron injection dynamics as a function of the position on the surface. (a) Map of electron injection. The time constants are extracted from TR-STML transients measured on a 10 × 7 grid (400 s integration time per pixel, U_on_ = −2.83 V, U_off_ = −2.53 V) and spatially interpolated. (b) Photon map of the same region as in panel a. An overlay of the C_60_ lattice at the interface (grid) is represented by dashed circles (lines). The color scale intensity ranges from 0 to 10 kcts/s. (c–f) Light intensity snapshots extracted at the indicated time delays after the arrival of the pulse to the junction. The images are normalized to the maximal light intensity over the whole data set, which is presented in Video S1. (g–j) Light intensity snapshots normalized to the maximum on each pixel reached after ca. 50 ns. The luminescence reaches its maximum faster at the center of the EC (lower part of the images). All scale bars are 1 nm.

The measurements described above can also be represented in a sequence of images showing the time evolution of light emission by slicing the 3-dimensional data block along constant delay times. At first, we compare the map of fitted steady-state intensity (Figure 3f) that is usually reached after 30–40 ns (see Figure 2d) with the photon map (Figure 3b) of the same area. Indeed, the snapshot reproduces correctly the spatial extent of the EC, measured by the spectrally integrated photon map. Next, we present the snapshots for various delays in Figure 3c–f, which are normalized to the highest recorded intensity within the whole data set. As expected from the lateral dependence of the charging time (Figure 2), the light intensity evolves slower when the tip is positioned at the periphery of the EC and is a direct visualization of the increase in the electron capture time. This time evolution is emphasized in Figure 3g–j, where each point has been scaled to its intensity maximum reached under steady-state conditions so that each pixel eventually reaches a value of 1. For instance, Figure 3h,i demonstrates that, in the central part of the EC (lower part of the images), the relative intensity is higher than at the peripheries (upper part of the images). By measuring the nanosecond-resolved light emission using TR-STML, we can thus probe and follow the single charge transfer time in 4 dimensions at the molecular scale with GHz frame rates (intervals of 0.7 ns) as visualized in Video S1.

The observed reduction in the electron injection rate (i.e., increase in the charge transfer time constant) as a function of the distance from the central position of the EC can be explained by the electric field inside the C_60_ film. The electron and hole trap states have the LDOS maximum in the center of the EC with the LDOS decaying with the lateral distance.^29^ Thus, when the tip is localized at the periphery of the EC, the hole can still reach the defect state and create a charged defect state EC^+^. Additionally, the probability of trapping the hole at the defect is reduced compared to the central positions of the EC, because it is more likely that the hole will be transported through the semiconducting C_60_ layer directly to the substrate. This results in a lower electroluminescence yield, as shown in Figure 2d (number of counts per time bin) and Figure 3b. The electron capture by EC^+^ is purely field-driven, does not involve transport from the tip, and thus constitutes a parameter independent of the emission intensity. It is induced at some distance away from the tip apex, similarly as the sharp rings observed in d*I*/d*V* maps which indicate local charging effects by the tip stray field.^7−10^ The electric field is controlled by the tip position as confirmed by electrostatic calculations (see the Methods section) shown in Figure 4. Remarkably, when the tip is located at the periphery of the EC, the electric field is reduced but still sufficiently strong at the EC^+^ position, allowing the electron to tunnel. In Figure 4c, we compare the potential energy situation for the cases in Figure 4a,b and find that the potential energy barrier for the electron injection from the substrate is increased by 0.1 eV (Δ*E*) when the tip is displaced 2.5 nm from the location of the hole. Δ*E* increases gradually when the tip is moved away from EC^+^ as plotted in Figure 4d, which slows down the exciton formation process (Figure 2e). A similar energy barrier increase is observed when the tip is retracted from the surface above the defect,^27^ as shown in Figure 2e.

**Figure 4:**
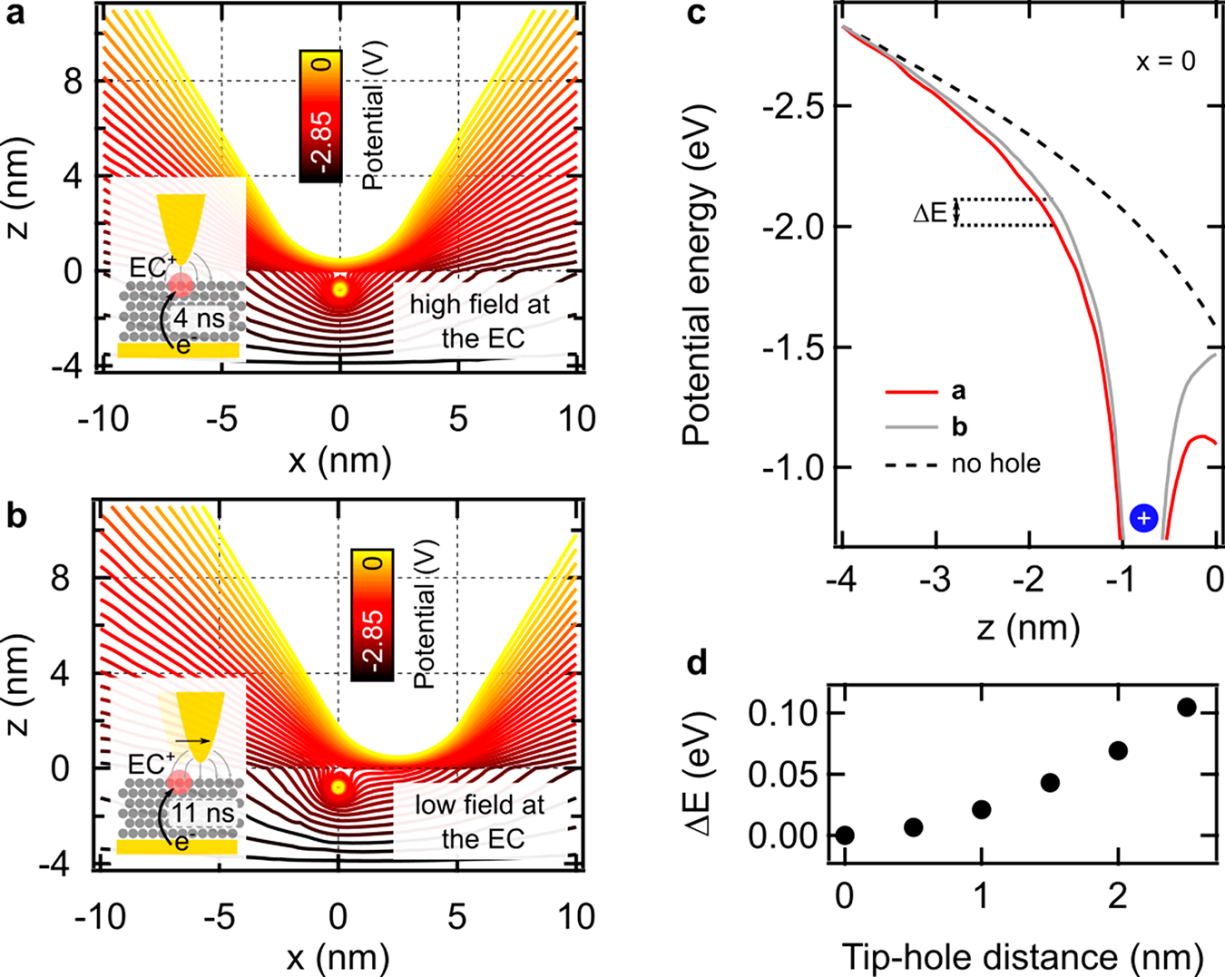
Electric potential at EC^+^ as a function of the tip position. Two limit cases are presented, the tip placed at the center of the EC (a) and 2.5 nm away (b). (c) Potential cross sections along the position *x* = 0. The dashed line shows the potential in the absence of the hole. (d) Increase of the potential barrier when the tip moves away from EC^+^.

Alternatively, one might assume that the exciton formation due to charge injection occurs always below the tip apex followed by exciton diffusion toward the defect where the exciton would radiatively decay. In that case, the observed increase of the time constant could be related to the diffusion time of the exciton. However, the diffusion process of the exciton occurs within its lifetime that is shorter than the resolution of the experiment for our ECs and thus cannot be resolved. Even though this scenario is consistent with the intensity falloff with distance from the defect, it is not compatible with the observed slowed-down dynamics at a large distance. Exciton formation away from the defect would rather require a constant or even shorter electron injection time since in this scenario the hole would not get trapped and rapidly move to the substrate—driven by the strong electric field. Finally, we can exclude that the increased nanosecond time delay results from the diffusion of the hole injected by the tip at some nanometer distance from the defect, as the charge hopping time in C_60_ was reported to be in the femtosecond regime.^35^

In conclusion, we establish an approach to image a light emitter at the nanoscale and map the evolution of its light emission with a rate of 10^9^ frames per second, an approach that goes beyond the steady-state submolecular mapping using STML. The observed luminescence evolution reflects the electron transfer from the substrate to a localized emitter and is controlled by the electric stray field of the STM tip, mimicking the energy landscape modifications induced by localized charges and different molecular species. While the overall charge transport in our system is dominated by the current passing through the HOMO-derived states, monitoring electroluminescence allows us to be sensitive to the electron injection to the defect only. We envision our approach to be used in future studies to explore single-electron injection dynamics that reach even submolecular resolution with single-molecule emitters or atomic point defects. In a material whose exciton lifetime is increased, for instance, based on triplet emission, the method presented here could be adapted to study exciton diffusion in real time.

## Methods

### Scanning Tunneling Microscopy-Induced Luminescence

All experiments were performed using a home-built ultra-high-vacuum low-temperature (4 K) STM with optical access provided by three lenses located in the STM head with their focus on the tip apex. We couple one of the resulting three independent light paths to a single-photon avalanche photodiode (SPADs, MPD-PDM-R) and another one to an optical spectrometer (spectrograph, Acton SP 300i; CCD camera, PI-MAX). The Au(111) crystal is prepared by repeated cycles of Ar^+^ sputtering and annealing (up to 850 K). C_60_ is thermally evaporated from a Knudsen cell (850 K) for 1 h on the crystal held at room temperature.

### Time-Resolved Measurements

Transmission function-corrected^27,32^ voltage pulses (2 MHz repetition rate, 100 ns length, 1 ns rise/fall time) are produced by an arbitrary wave generator (AWG, Agilent M8190A) and sent to the tunnel junction through high-frequency optimized wiring (semirigid and coaxial cables). The amplitude of the pulses is −300 mV, which is added to the DC offset bias (*U*_off_) by a bias tee (Picosecond Pulse Laboratories, 5550B). *I*_pulse_ is defined as the tunneling current measured at *U*_on_ for the same tip–sample distance as during the pulse measurement. For the measurements shown in Figure 3, the feedback loop was off during acquisition of the transient but turned on between the measurements to correct for the *z* drift of the STM tip. To minimize the overall drift, the tip was stabilized at the EC for 10 h before the series. The integration time per point was 400 s. More details on the measurement can be found in the Supporting Information.

### Electrostatic Calculations

The calculations are done with Mecway finite element analysis software (Mecway Ltd.) in the full 3D geometry of the problem. The results are represented in the figures by a cut along the symmetry plane of the geometry defined by the tip axis and the position of the charge. For details, see the Supporting Information.

## Supporting Information Available

Size of the emission centers, details of the characterization of the single electron injection dynamics in Figure 2, details of the grid measurement, electrostatic potential calculations, and supporting references (PDF)
Video S1: evolution of sub-nm electroluminescence in 0.7 ns intervals (AVI)

## Supplementary Material

nl1c00328_si_001.pdf

nl1c00328_si_002.avi
