# Supplementary material for: Gigahertz Frame Rate Imaging of Charge-Injection Dynamics in a Molecular Light Source
Source: Nano Lett. 2021 May 26;21(11):4577–83. doi: 10.1021/acs.nanolett.1c00328 (PMC8193635; doi:10.1021/acs.nanolett.1c00328)
Supplement: Supplementary file 1 — nl1c00328_si_001.pdf [file nl1c00328_si_001.pdf]

Supporting information for:

Gigahertz frame rate imaging of charge-injection dynamics in a molecular light source

Anna Rosławska[1,2,*], Pablo Merino[1,3,4], Christopher C. Leon[1], Abhishek Grewal[1], Markus Etzkorn[1,5], Klaus Kuhnke[1,*], Klaus Kern[1,6]

[1] Max-Planck-Institut für Festkörperforschung, D-70569, Stuttgart, Germany.

[2] Université de Strasbourg, CNRS, IPCMS, UMR 7504, F-67000 Strasbourg, France.

[3] Instituto de Ciencia de Materiales de Madrid, CSIC, E-28049, Madrid, Spain.

[4] Instituto de Física Fundamental, CSIC, E-28006, Madrid, Spain.

[5] Institut für Angewandte Physik, TU Braunschweig, D-38106 Braunschweig, Germany.

[6] Institut de Physique, École Polytechnique Fédérale de Lausanne, CH-1015 Lausanne, Switzerland.

* roslawska@ipcms.unistra.fr

* k.kuhnke@fkf.mpg.de

Size of the emission centers

For defects located in the subsurface layers, the apparent size (bright area on the surface) of the emission center (EC) can be even larger than the size of the ECs located at the top layer. Already for such an EC, even though the local density of states (LDOS) of the hole trap state is the highest in the central area of the EC, there is still an experimentally measurable increase in the LDOS signal at a lateral distance of 2-3 nm from the center[1]. In the case of a subsurface defect, a hole transported through the film may hop sideways due to the hexagonal stacking, resulting in a larger efficient lateral distance from which the hole can be injected into the defect. Fig. S1 shows such variation in sizes of the ECs, which can have a diameter ranging from 1-2 molecules to 5-6.

The emission centers selected for our study are chosen such that they are spatially isolated from other ECs and show an individual emission line, followed by its vibrational fingerprint, such as the ones presented in Fig. 2c and Fig. S8b. This well-defined energy of the transition arises due to the electronic states of the studied defect and thus varies from EC to EC[1,2]. Indeed, the EC studied in Fig. 2 is characterized by 1.80 eV emission line (Fig. 2c), while the EC studied in Fig. 3 (Fig. S8b) emits at 1.78 eV.

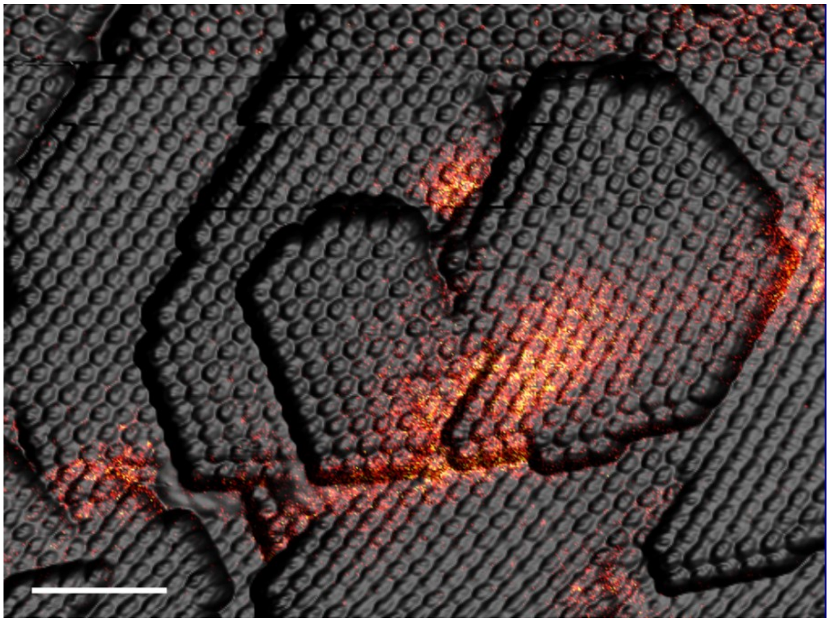

Figure S1. Topography overlaid with electroluminescence yield map. U = - 3 V, I = 30 pA. Scale bar 5 nm. Intensity range (0-3 kcts s^{-1}).

Details of the characterization of the single electron injection dynamics in Fig. 2

Origin of the EC

The origin of the EC presented in Fig. 2 of the main text may be related to a defect located on the surface or in one of the top layers of the C_{60} film. Some of the ECs are related to a molecular misorientation (rotation) of molecules in the low-temperature C_{60} (2x2) orientational superstructure[1], which is indicated in Fig S2a (the same area as in Fig. 2a in the main manuscript). In the case presented in Fig. 2, there are some perturbations in the superstructure in positions slightly off-center of the EC as marked in Fig. S2a by red dots. They are, however, not located at the central position where the defect trapping the charges and excitons is expected to be[1]. Thus, it is likely that the defect leading to split-off states is located below the surface. It is further supported by the fact that there are no split-off states visible in the scanning tunneling spectroscopy at position 1 (Fig. S2b) and only the onsets of the C_{60} bands are resolved.

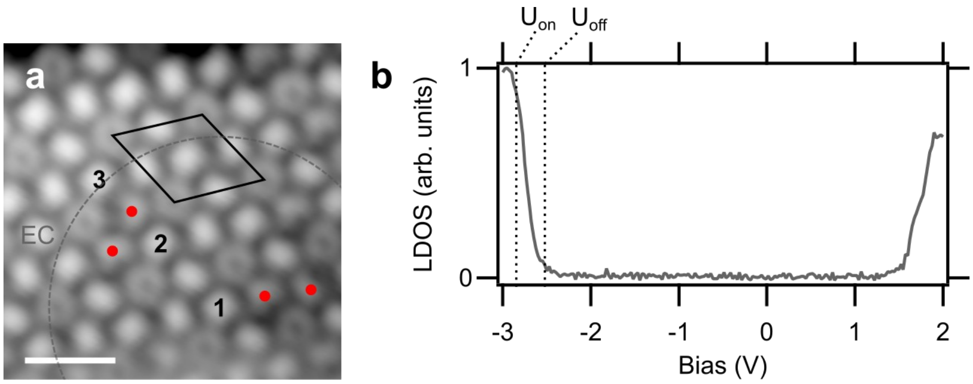

Figure S2. Detailed characterization of the EC. a) STM constant current topographic scan of the same area as in Fig. 2a of the main text, U = -3 V, I = 30 pA. The spatial extent of the EC is marked by a dashed circle, the (2x2) superstructure is marked by a black rhombus, red circles mark the molecules that have a perturbed orientation. Scale bar 2 nm. b) Scanning tunneling dI/dV spectrum measuring the LDOS at position 1 in Fig. S2a, set-point: U =- 3 V, I = 30 pA.

Vertical and lateral dependence of the dynamics

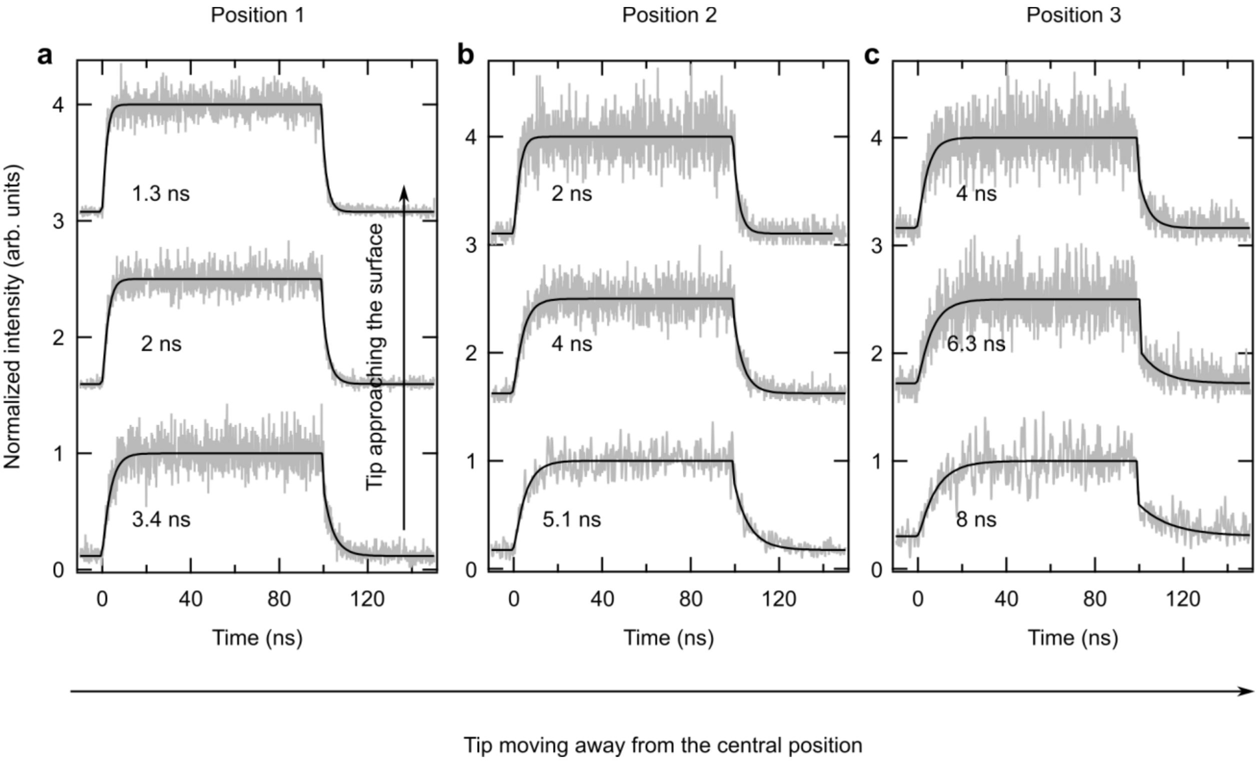

Figure S3. TR-STML transients used to extract the information presented in Fig. 2e of the main text. The solid lines are the fits to the model. The charges are injected faster when the tip is located closer to the defect both vertically and laterally.

Single-electron injection dynamics extracted from the falling edge of the transients

Fig. 2e of the main manuscript presents the time constants of the electron injection (τ_e) extracted from the rising edge (approx. first 20 ns) of the transients. The fits to the kinetic model also yield the τ_e embedded in the falling edge of the transient, which is plotted in Fig. S4. Similar to the tendency observed in Fig. 2e, the electron injection is slower when the tip is located at the peripheries of the EC. The time constants are longer for the falling edge because the bias (thus, the electric field) is lower and the electron injection is less favorable. We note that for the measurements presented in this work the voltage between the pulses (U_{off}) is already close to the onset of the highest occupied molecular orbital (HOMO) band, meaning that hole injection and light emission may take place between the pulses[3]. However, the

emission intensity for t < 0 ns and t > 150 ns is very low and does not affect the exponential fit and the obtained time constant.

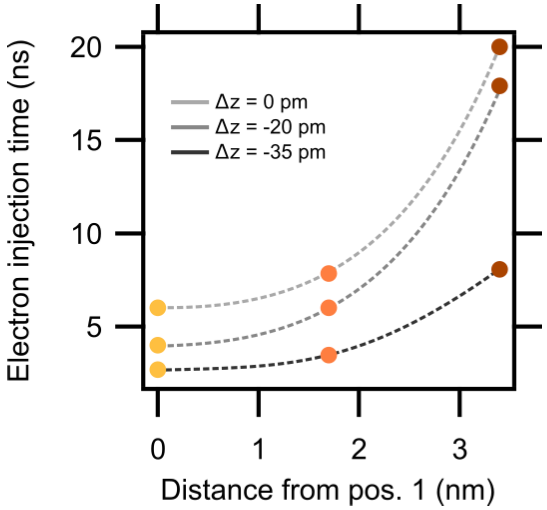

Figure S4. Electron injection time extracted from the falling edges of TR-STML transients recorded at positions 1-3 marked in Fig. S2a. I_{pulse} = 12 pA (Δz = 0 pm), I_{pulse} = 20 pA (Δz = -20 pm), I_{pulse} = 37 pA (Δz = -35 pm), U_{on} =- 2.83 V, U_{off} = -2.53 V. The dashed lines are guides to the eye.

Details of the grid measurement (Fig. 3)

The measurements presented in Fig. 3 have been performed on a 10x7 rectangular grid corresponding to an area of 5x3.7 nm^2 with an integration time 400 s per pixel, 8 h for the whole data set. The three last points of the grid (upper right corner) are discarded due to a tip change (likely picking up a C_{60} molecule from the layer) and replaced by the value of the last point before the change occurred. This operation preserves the spatial characteristics of the dynamics as evidenced in Fig. 3a of the main text.

Fig. S5 shows two traces demonstrating two representative cases from the dataset of the registered dynamics together with the fits to the kinetic model yielding electron injection time constants of 4.8 ns and 12 ns respectively.

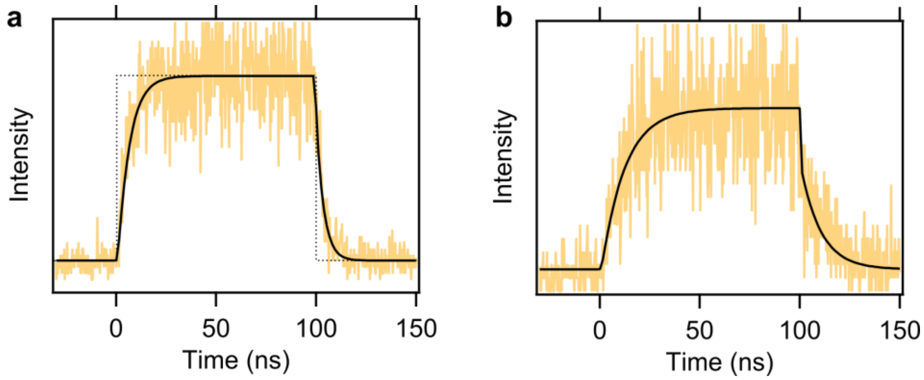

Figure S5. Two representative transients obtained within the grid measurement. The yellow trace is the raw data; the black curve is the fit to the kinetic model. a) τ_{el} *= 4.8 ns b)* τ_{el} *= 12 ns. The dotted line in a) indicates the shape and sharpness of the applied voltage pulse.*

The electron injection dynamics is present also in the falling edge (100-120 ns) of the transient. In that situation, the hole captured in the defect when the voltage pulse was present in the junction remains after the voltage returns to a value inside the bandgap. The hole shifts the electron trap sufficiently below the Fermi level of the substrate such that the electron can be still injected, and its dynamics mapped. Such a map is presented in Fig. S6b and shows a similar spatial dependence in which the measured time constant is slower in the peripheries of the EC. Due to the lower electric field (lower absolute bias voltage when the pulse is over), the time constants are relatively longer as compared to the rising edge (Fig. S6a). However, in that case, the electron injection dynamics is convoluted with another transport process in which the hole may tunnel to the tip (hole detrapping). It occurs on a similar time scale[3] and reduces the dynamical contrast. Therefore, the time constant map extracted from the rising edge (0-20 ns) of the

transients (Fig. 3a) provides a more direct measure of the single electron injection dynamics. For a clear comparison, we show the maps in a raw, non-interpolated form (Fig. S6).

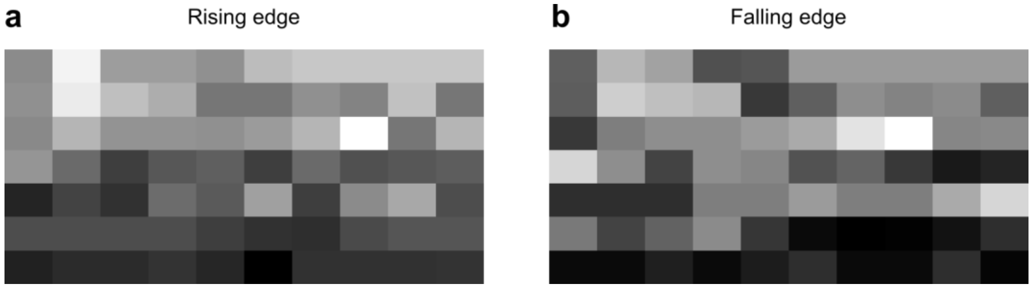

Figure S6. Comparison between the electron injection time constant extracted from the rising (a) and falling (b) edges of the pulse. The studied area is the same as in Fig. 3 of the main text. Time constant ranges (black-white color scale): a) 3 ns – 13 ns. b) 7.1 ns – 25 ns.

To correct for the z drift, we enable the feedback loop in-between measurements, which may result in a slight variation of tip-sample distance and affect the measured dynamics, similar to the data presented in Fig. 2e of the main text. To verify that our approach is correct we plot the relative z displacement at every position and present it in Fig. S7. Comparing Fig. S7 with Fig. S6 yields no correlation, so the variations in the tip-sample distance do not affect the general trend of the dynamics (in periphery of the EC the dynamics are slower). We note that these variations as well as to local changes in the topography, electronic structure, the non-symmetric tip shape may be responsible for the local non-monotonicity of the trend as visible in Fig. 3a and Fig S7a.

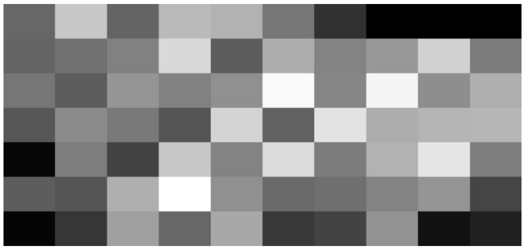

Figure S7. Z displacement during the measurement. The studied area is the same as in Fig. 3 of the main text. Relative displacement range (black-white color scale): 0 – 115 pm.

In Supporting Video 1, we show a continuous evolution of the snapshots presented in Fig. 3: an “ns-nm video” covering the rising edge of the transients with subsequent snapshots every 0.7 ns. The video illustrates the evolution of light emission as a function of the tip position and is a real-time measure of the electron injection dynamics.

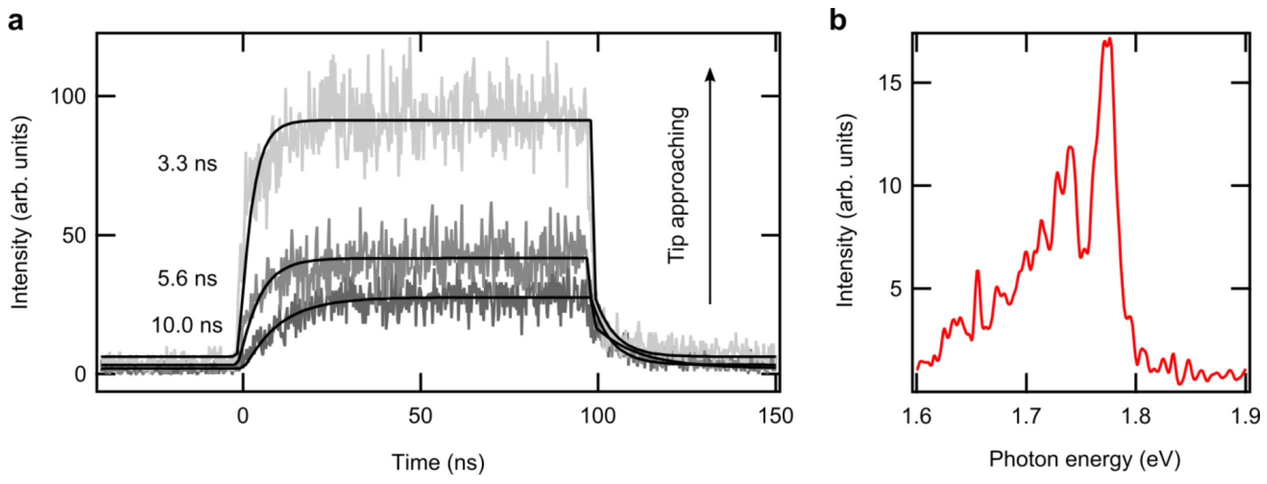

Figure S8. a) TR-STML transients recorded at the EC presented in Fig. 3 of the main text as a function of the tip-sample distance. The values of the current during the pulse, I_{pulse} were 3.4, 1.4, 0.9 pA (from top to bottom respectively). U_{on} =- 2.83 V, U_{off} = -2.53 V. The electron injection time constants are indicated next to the graphs and follow the trend presented in Fig. 2 of the main text and Fig. S3. b) Optical spectrum, U = - 3 V, I = 30 pA, t = 600 s.

For consistency, Fig. S8a shows the TR-STML transients recorded at this EC as a function of the tip-sample separation that show the expected trend of increased dynamics when the tip approaches the EC. Fig. S8b shows an STML spectrum of the probed EC showing a single exciton line followed by vibrational progression.

Electrostatic potential calculations for a charge in the C_{60} layer below the STM tip

Electrostatic calculations were performed on a discrete grid using the Mecway finite element analysis software [Mecway Ltd., New Zealand] in the full 3D geometry of the problem making use of the mirror plane defined by the tip axis and the charge position which allows reducing the calculation to one half space. The grid consists of a total of 34367 elements with unevenly spaced points with the point density substantially increasing towards the tunnel junction and the charge position (see Fig. S9). The tip electrode consists of 1440 points set to 0 V potential with a conical shape (opening angle 60°) merged with a spherical shape of radius 3 nm. The distance from the tip apex to the plane C_{60} surface is 0.5 nm, the thickness of the C_{60} film (dielectric constant ε=4.4) is 4.0 nm. The elementary charge (Q = 1.6e-19 C) is placed at 0.8 nm depth below the surface inside the C_{60} film. The plane substrate electrode below the C_{60} film is defined by 688 points set to a potential of -2.85 V. For the presentation of the results potential values on the point charge and on its directly neighboring points have been removed as they tend to exhibit artefacts.

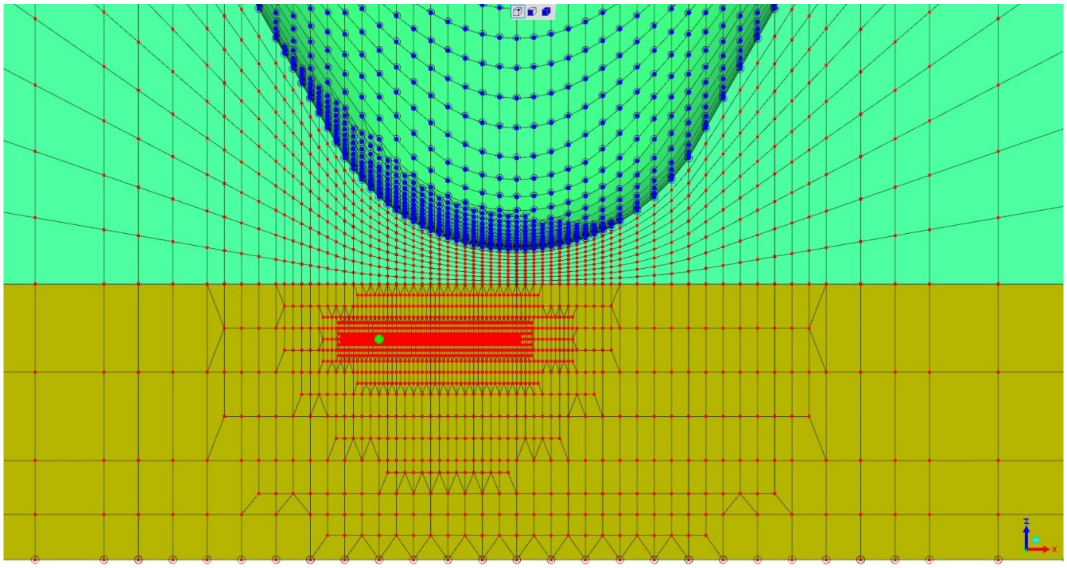

Figure S9. Mecway plot of the 2-dimensional projection of the discrete grid for the calculation of the electrostatic potential in the STM junction with an elementary point charge (green dot) buried within the C_{60} film. The projection reveals the complete grid of the (hollow) tip electrode.

Supporting references

1. Große, C., Gunnarsson, O., Merino, P., Kuhnke, K. & Kern, K. Nanoscale Imaging of Charge Carrier and Exciton Trapping at Structural Defects in Organic Semiconductors. *Nano Lett.* **16**, 2084–2089 (2016).
2. Große, C. *et al.* Submolecular Electroluminescence Mapping of Organic Semiconductors. *ACS Nano* **11**, 1230–1237 (2017).
3. Rosławska, A. *et al.* Single Charge and Exciton Dynamics Probed by Molecular-Scale-Induced Electroluminescence. *Nano Lett.* **18**, 4001–4007 (2018).
